# MRI in perianal fistulae

**DOI:** 10.4103/0971-3026.59756

**Published:** 2010-02

**Authors:** Pushpinder S Khera, Hesham A Badawi, Ahmed H Afifi

**Affiliations:** Department of Clinical Radiology, Al Sabah Hospital, Kuwait City, Kuwait, Medical Research Institute, Alexandria University, Egypt; 1 Department of Radiology, Medical Research Institute, Alexandria University, Egypt

**Keywords:** Perianal fistulae, MRI, intersphincteric, transsphincteric

## Abstract

MRI has become the method of choice for evaluating perianal fistulae due to its ability to display the anatomy of the sphincter muscles orthogonally, with good contrast resolution. In this article we give an outline of the classification of perianal fistulae and present a pictorial assay of sphincter anatomy and the MRI findings in perianal fistulae. This study is based on a retrospective analysis of 43 patients with a clinical diagnosis of perianal fistula. MRI revealed a total of 44 fistulae in 35 patients; eight patients had only perianal sinuses.

## Introduction

Perianal fistulae commonly occur in middle-aged men.[1] They are thought to be a result of anal gland obstruction, with secondary abscess formation and external rupture of the abscess.[1] They have traditionally been imaged by conventional fistulograms; the procedure involves cannulation of the external opening and injection of a water-soluble contrast into the fistula. This method has two main disadvantages: First, the primary tract and its extensions do not fill with contrast if they are plugged with pus or debris and, second, the sphincter muscle anatomy is not imaged and hence the relation between the tract, the internal/external sphincter, and the levator ani muscle is not revealed.[2]

Transrectal ultrasound better depicts fistulae and their relation to the anal sphincter muscles. The operator dependence, limited field of view and absence of a coronal plane of imaging, however, are its disadvantages.[2]

CT fistulography is limited by the fact that attenuation values of the fistula tract, the areas of fibrosis, and sphincter muscles are similar to each other.[2] Multidetector row CT fistulography with its isotropic voxels is expected to improve the results from this modality.[2]

The role of MR fistulography in the preoperative evaluation of perianal fistulae is now well established.[3–5]

### Normal MRI anatomy of the anal sphincter [Figure 1]

**Figure 1 (a-e):**
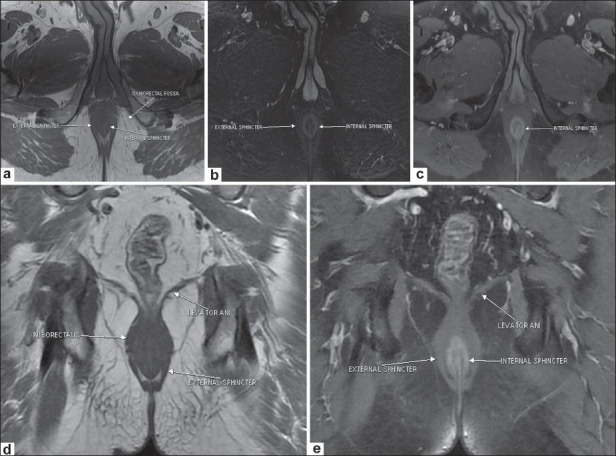
Normal MRI anatomy of the sphincters. Axial T1W image (a) shows the internal and external sphincters. Axial fat-saturated T2W image (b) shows the relatively hyperintense internal sphincter bounded laterally by the hypointense external sphincter. Postcontrast axial T1W image (c) depicts normal enhancement of the internal sphincter. Coronal T1W image (d) delineates the levator ani. Coronal postcontrast T1W image (e) shows the internal and external sphincters and the levator ani

An optimal examination utilizes both endoluminal and external phased-array surface coils.[2] However, imaging with an external coil alone also provides good results.[4,6,7] The external anal sphincter (a striated muscle) is clearly visualized on MRI. It is hypointense on T1W, T2W, and fat-suppressed T2W images, and is bordered laterally by the fat in the ischioanal fossa [Figure 1 a,b and d].

The internal sphincter (a smooth muscle) is hypointense on T1W [Figure 1a] and T2W TSE images and is relatively hyperintense on fat-suppressed T2W images [Figure 1b]. It shows enhancement on post-gadolinium T1W images[2] [Figure 1c].

The coronal images depict the levator ani muscle (levator plane), the identification of which is important to distinguish supralevator from infralevator infections. The puborectalis ring is seen as a thickening of the superior fibers of the external sphincter [Figure 1d]. The puborectalis further merges with the levator plate superiorly.

### Classification of perianal fistulae

Depending on the location and course of the primary tract, perianal fistulae have been classified into four types.[8]

1. Intersphincteric (incidence 60-70%):[9] The infection starts from an anal gland and develops in the inter sphincteric plane, lying between the internal and external sphincters, without penetrating the external sphincter. It eventually ruptures onto the skin, thereby creating the fistula.
2. Transsphincteric (incidence 20-30%):[9] This occurs when the intersphincteric infection penetrates the external sphincter to reach the ischioanal fossa and, eventually, the perianal skin.
3. Suprasphincteric (uncommon): These fistulae extend superiorly in the intersphincteric plane to reach above the levator plane and then penetrate inferiorly through the ischioanal fossa.
4. Extrasphincteric (uncommon): These result from extension of primary pelvic disease (e.g., Crohn's disease, diverticulitis, radiation proctitis) down through the levator plate.

## Materials and Methods

The study population comprised 43 patients whose MRI studies (done between October 2002 and December 2008) were evaluated retrospectively. The patients had been referred to the MRI unit for MR fistulography.

All MRI studies were carried out on a 1.5-T MRI system (GE Signa Excite 1.5T) using an 8-channel phased-array coil. The sequences evaluated were: Axial T1 TSE (TR/ TE 700 – 750/8 −15, FOV 24 × 24, matrix 250 × 224, Nex 2); Axial fat-suppressed T2W (TR/TE 3500 – 4000/102, FOV 24 × 24, matrix 250 × 224, Nex 2); Axial post-contrast T1W [post injection of 10 ml of gadodiamide (Gd-DTPA-BMA), TR/TE 540/10, FOV 256 × 352, matrix 24 × 24, Nex 2]; Coronal T1W TSE (TR/TE 540/8, FOV 24 × 24, matrix 352 × 256, Nex 2); Coronal T2W fat-suppressed (TR/ TE 2900/98, FOV 24 × 24, matrix 325 × 256, Nex 2); and coronal post-contrast T1W (TR/TE 540/10, FOV 24 × 24, matrix 256 × 352, Nex 2).

## Results

Of the 43 patients in our study, eight (18%) were identified as having a perianal sinus only, with no fistula extending into the anal canal. The rest of the 35 cases were evaluated for the site of the primary tract and its ramifications, the presence/absence of external sphincter involvement, and the location of the internal openings.

**Figure 2 (a-e):**
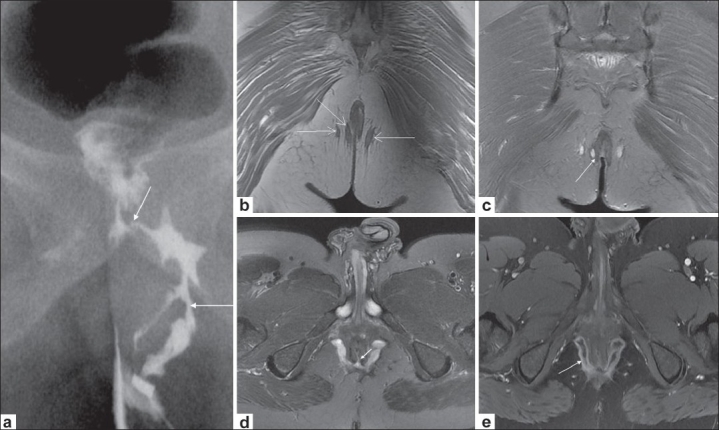
Crohn's disease with extrasphincteric fistulae. Conventional fistulogram (a) shows a branching left perianal fistula (long arrow), communicating with the rectum (short arrow). Coronal T1W image (b) depicts bilateral ischiorectal tracts (arrows). Coronal fat-saturated T2W image (c) depicts one transsphincteric tract interrupting the right external sphincter (arrow), the two other tracts being extrasphincteric. Axial fat-saturated T2W image (d) shows bilateral ischiorectal abscesses communicating across the midline with a posterior internal opening at 6 o'clock position (arrow). Axial postcontrast T1W image (e) depicts enhancement of the horseshoe abscess (arrow)

Three patients had a primary or recurrent perianal fistula with associated Crohn's disease [Figure 2]. Two of these three cases had multiple fistulae and all three had abscess formation [Figure 2d and e]. Of the remaining 32 patients without Crohn's disease, 24 had a primary fistula and, of these, seven had previously undergone perianal abscess drainage. Eight patients had undergone previous fistula surgery and had presented with a recurrence.

**Figure 3 (a-e):**
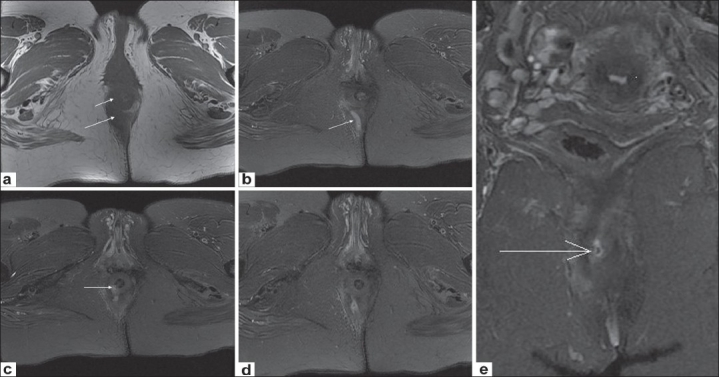
Transsphincteric fistula. Axial T1W image (a) shows inflammation in the right ischiorectal fossa (long arrow) and thickening of the right external sphincter (short arrow). Sequential caudocranial axial fat-saturated T2W images (b-d) depict a hyperintense tract in the right ischiorectal fossa (arrow in b) and its penetration of the right external sphincter (arrow in (d). Coronal fat-saturated T2W image (e) also shows the internal rectal opening (arrow)

**Figure 4 (a-d):**
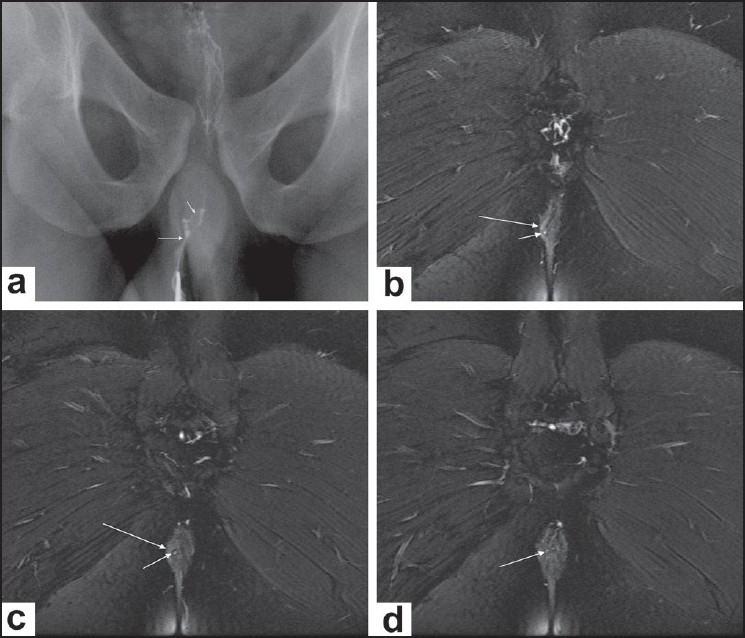
Intersphincteric fistula. Conventional fistulogram (a) delineates a thin tract lying (long arrow) close to the midline, with internal communication (short arrow). Sequential posterior to anterior coronal fat-saturated T2W images (b-d) reveal a small hyperintense tract (short arrow in b,c) lying entirely medial to the right external sphincter (long arrow in b, c) and opening within the anal canal (arrow in d)

**Figure 5 (a-e):**
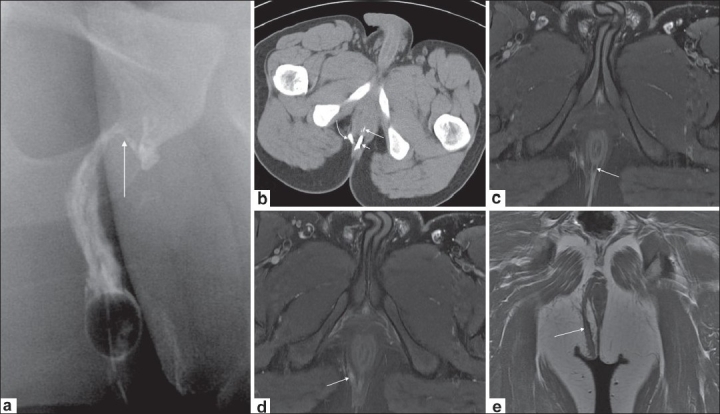
Combined trans- and extrasphincteric fistula. Conventional fistulogram (a) shows a right-sided large well-defined fistula (arrow). CT fistulogram (b) depicts contrast in the rectum (long arrow) with two closely located tracts, one lying close to the midline (short arrow) and the other being lateral (curved arrow) to the right external sphincter (extrasphincteric). The axial fat-saturated T2W images (c and d) reveal one tract to be transsphincteric (arrow in c) and the other to be extrasphincteric (arrow in d). Coronal T1W image (e) depicts the complete course of the extrasphincteric tract (arrow)

Out of a total of 44 fistulae in these 35 patients, 14 (33%) were transsphincteric [Figure 3], 25 (60%) were intersphincteric [Figure 4] and three (7%) were extrasphincteric [Figure 5]. No suprasphincteric fistula was encountered in the study. Twenty-seven fistulae (61%) were simple, whereas 17 (39%) showed complications (abscess formation, branching course, inflammatory tissue, etc).

## Discussion

MRI imaging of perianal fistulae relies on the inherent high soft tissue contrast resolution and the multiplanar display of anatomy by this modality. In one of the early studies on MRI fistulography, Lunniss *et al*. reported a concordance rate of 86-88% between MRI and surgical findings.[10] Subsequent studies have suggested that MRI is more sensitive than even surgical exploration of the tract.[5,11] MRI is especially useful in patients with fistulae associated with Crohn's disease and those with recurrent fistulae,[3] as these entities are associated with branching fistulous tracts. Missed extensions are the commonest cause of recurrence.[12]

T2W images (TSE and fat-suppressed) provide good contrast between the hyperintense fluid in the tract and the hypointense fibrous wall of the fistula, while providing good delineation of the layers of the anal sphincter.[6,13] In our experience, axial T2W fat-suppressed images were the most useful for locating the fistulous tract.

Gadolinium-enhanced T1W images are useful to differentiate a fluid-filled tract from an area of inflammation.[14] The tract wall enhances, whereas the central portion is hypointense. Abscesses are also very well depicted on post-gadolinium images.

The exact location of the primary tract (ischioanal or intersphincteric) is most easily visualized on axial images; the presence of disruption of the external anal sphincter differentiates a transsphincteric fistula from an intersphincteric one. The internal opening of the fistula is also best seen in this plane.

As mentioned earlier, coronal images depict the levator plane, thereby allowing differentiation of supralevator from infralevator infection. A combination of an axial and a longitudinal series (coronal, sagittal, or radial) will provide all the necessary details.[15]

To summarise, evaluation of an enhanced T1W image, in conjunction with a fat-suppressed T2W image, provides most of the details necessary for accurate evaluation of perianal fistulae.
